# A Qualitative Study of a New Generation of Irish-Trained Doctors’ Views on Selection for Medicine

**DOI:** 10.12688/mep.21457.1

**Published:** 2026-01-26

**Authors:** Seán Barber, Deirdre Bennett

**Affiliations:** 1 University College Cork School of Medicine, Cork, County Cork, Ireland

**Keywords:** Medical student selection, stakeholder opinions, HPAT-Ireland, aptitude testing, assessment validity, equity and access, qualitative research, thematic analysis

## Abstract

**Background:**

Student selection for Medicine degree programmes is a complex and high-stakes process. In Ireland, students entering from secondary school are selected based on combined scores achieved in the state school exit examination (Leaving Certificate) and the Health Professions Aptitude Test (HPAT-Ireland), introduced in 2009 to broaden entry assessment beyond academic achievement alone. Questions remain about the validity, fairness, and alignment with the skills required for medical practice of this approach. This study explored the views of the “HPAT generation” (doctors admitted via HPAT-Ireland) on ideals and processes of medical student selection, including the relevance and equity of the Irish system.

**Methods:**

An interpretivist qualitative study using semi-structured interviews was conducted and analysed using reflexive thematic analysis.

**Results:**

Twelve Irish-trained doctors admitted post-2009 were interviewed. Four themes were identified. Participants identified *Threshold criteria as foundations of selection* (Theme 1) highlighting academic ability and motivation as essential, with the Leaving Certificate viewed as an appropriate threshold measure. They also identified *Further criteria as top-level differentiators* (Theme 2) including problem-solving, critical thinking, and communication skills. Interviews, multiple mini-interviews, and selection centres were seen as authentic assessments albeit limited by bias and nepotism. HPAT-Ireland was criticised for limited scope, artificial format, and socioeconomic inequity. Under *Personal diversity and potential for development* (Theme 3) participants emphasised varied personalities, and recognition that desirable qualities can be cultivated during training. Under *Equity, access, and exploitation* (Theme 4) participants expressed concerns that any selection method may be prone to financial inequity and exploitative score inflation without systems-level change.

**Conclusions:**

Findings highlight tensions between academic thresholds, authentic assessment of differentiators, and equitable access. Doctors of the HPAT-Ireland generation support a two-stage selection system but are sceptical of HPAT-Ireland’s ability to fulfil its intended role. Policy reforms must address these tensions to ensure fair and sustainable medical student selection.

## Introduction

Student selection for undergraduate medical degree programmes is a high-stakes process, with significant variation in selection methods across countries ^1, 2^. Common methods include academic assessment, aptitude tests, interviews (traditional, multiple mini interviews (MMIs), or selection centres (SCs)), situational judgement tests (SJTs), and portfolio or essay submissions ^1, 2^. Recent evidence suggests that academic achievement is the strongest predictor of medical school success, while MMIs, SJTs, and SCs provide moderate validity in assessing non-cognitive attributes ^1, 3– 5^. Aptitude tests are seen to have mixed results: reasoning and problem solving components show some predictive value but interpersonal domains are less reliable ^3, 6, 7^. Importantly, most research evaluates prediction of academic performance rather than graduate outcomes ^1^, leaving questions about whether these tools identify the qualities most important in clinical practice.

In Ireland, undergraduate selection combines the state level standardised secondary school/high school examination, the Leaving Certificate (LC), with HPAT-Ireland, a 3-hour multiple choice question aptitude test introduced in 2009 to broaden assessment beyond purely academic achievement, specifically to domains of [1] logical reasoning and problem solving, [2] interpersonal and emotional understanding, and [3] non-verbal reasoning ^8^. Previously, reliance on LC alone favoured students from high-achieving schools and affluent families, reinforcing homogeneity and limiting diversity ^9^. HPAT was intended to mitigate these inequities by assessing the above skills – idealised as untrainable at the time of introduction ^10^. HPAT-Ireland contributes a maximum of 300 points to applications to medicine. While the Leaving Certificate (LC) is scored out of 625, medical applicants generally score above 550 (88th centile or higher in 2025 ^11^), and these scores are moderated such that each additional 5 LC points contributes 1 application point, up to a maximum LC contribution of 565 points.

There is evidence to suggest that HPAT has shifted the successful applicant cohort away from only ultra-high LC performers ^9^. Studies assessing its predictive validity, or whether it effectively forecasts clinical performance, empathy, or communication skills, have found limited or inconsistent evidence of a meaningful correlation ^7, 12^. As with international methods, research has focused on prediction of academic performance rather than professional performance or aptitude.

While research on HPAT-Ireland is limited, there is extensive research on the UMAT – a closely related aptitude test, assessing the same parameters, previously used in Australia and New Zealand ^13^. Findings consistently showed the most significant predictive power of academic assessment, moderate predictive power of logical reasoning/problem solving assessment, and mixed or negative predictive power of emotional and spatial reasoning ^14, 15^. Overall, UMAT only contributed slightly to prediction of late stage medical school performance and not at all to postgraduate performance ^16^. Moreover, the influence of practice and resits in inflating scores was clearly demonstrated ^17^. The UMAT has since been phased out in Australia, in favour of the UCAT, a broader assessment frequently used in conjunction with interviews ^18^.

Alongside predictive validity, the concept of political validity – how stakeholders perceive a tool’s fairness, appropriateness, and transparency, as defined by Kelly et al – is critical ^10, 19^. Political validity has been shown to influence the long-term sustainability of selection tools: if a test is viewed as biased, inaccessible, or unrepresentative of real-world competence, it risks undermining trust in the broader admissions process ^10^. Political validity is particularly relevant in the Irish context because HPAT-Ireland was introduced amid strong public and political pressure to broaden access to medicine programmes and to address perceived inequities and reliance on rote-learning in Leaving Certificate-based selection ^10^. Understanding whether the tool retains legitimacy with those who experienced it as applicants is therefore important for policy acceptance and sustainability.

Fifteen years after HPAT’s introduction, a new generation of doctors have been selected via this test. For the purposes of this paper, they will be termed the “HPAT generation”. This constitutes the only group with lived experience of both the selection tool and subsequent medical training. They offer unique retrospective insights unavailable from applicants, policymakers, or educators alone. As practising clinicians, their views integrate real-world experience of what attributes matter in training and practice, providing legitimacy to their evaluations of selection tools ^20^.

To date, few studies have assessed the views of stakeholders on Irish selection. Those that exist highlight inconsistent acceptability of aptitude tests, with recurring concerns regarding construct validity, predictive validity, and gender or socio-economic equality ^19, 21, 22^. To the knowledge of the authors, no studies have explored the view of the “HPAT generation”. Understanding their perceptions, alongside international comparisons (e.g. MMIs, interviews, personal statements), may help determine whether current processes align with values and competencies most important to current and future clinicians.

This work is particularly timely. Recent years have seen renewed calls for reform, fuelled by concerns about coaching, repeated sittings, and equity ^23^. In July 2025, medical school deans announced reforms to begin in 2027, including halving HPAT-Irelands’s contribution from 300 to 150 points, and removing high LC score moderation ^24^. These changes aim to reduce over-reliance on aptitude testing, reflect widening access schemes introduced since 2009, and align LC policy with international norms where score moderation is not typical ^24^.

The aims of this study are to explore how this HPAT generation perceive:

1) The attributes by which medical students should be selected.
2) The relevance, fairness, and validity of national and international selection methods.
3) The alignment of selection for medicine in Ireland with these principles, as informed by their lived experience.

In line with these aims, the specific research question to be answered is: What views regarding medical school selection ideals and processes, including that used in Ireland, are held by the generation of doctors who underwent HPAT-Ireland?

## Methodology and methods

### Research design

This study utilised a qualitative, interpretivist design – selected in line with the study’s aims to understand potentially complex, individual experience-based perspectives that could not be meaningfully quantified ^25^. The choice of paradigm informed data collection and analysis methods such as inductive coding and flexible interview structures. Data were collected through semi-structured interviews and analysed using reflexive thematic analysis in line with Braun and Clarke’s framework ^26^.

### Participants and sampling

The target population for this study was any currently practising doctor in Ireland or abroad, who studied medicine in Ireland, who completed HPAT-Ireland as part of their medical school application process.

Convenience and purposive sampling were used to ensure inclusion across gender, specialty, seniority, and geography, supplemented by snowball sampling ^27, 28^.

Selected individuals were contacted by email, or initially through personal and professional networks followed by formal email contact. Contact included a participant information leaflet, comprising a description of the proposed research and aims, details on the interview process, and a description of how data was to be handled, analysed, and reported. Also included was a consent form which agreeing participants digitally signed and returned. Written informed consent for the recording, analysis, and publishing of participants’ statements was obtained from the participants.

Recruitment was open-ended, and ceased when data sufficiency was deemed by the researchers to have been reached, indicated by diminishing novelty in the final 2 – 3 interviews, where no substantively new codes or perspectives were identified ^29^.

This research was not site specific. Most participants were based in Cork, Ireland, or Victoria, Australia, reflecting the researchers’ networks.

### Data collection

Data collection occurred between 09/05/2025 and 07/09/2025. Semi-structured interviews were conducted via Microsoft Teams to support guided yet flexible exploration of the research topic. An interview guide was developed from the literature, piloted, and iteratively refined during data collection to align with the emerging analytic focus. Interviews were conducted individually, audio-recorded with consent, and transcribed verbatim following automated transcription and manual verification. The final interview guide is provided as extended data (File 1).

### Ethics and data management

Ethical approval was obtained from the University College Cork (UCC) Social Research Ethics Committee (SREC) on March 6th 2025, with log number 2025-008. Data were collected and stored securely, with access restricted to the research team, and were anonymised prior to analysis. Qualitative data were analysed using NVivo. Due to ethical and confidentiality constraints, the data are not publicly available.

### Data analysis

Interview transcripts were managed in NVivo and analysed using reflexive thematic analysis, following Braun and Clarke’s six-phase framework ^26^:

1. Familiarisation was achieved through repeated reading, listening to recordings, and memo-writing.
2. Inductive latent coding was then undertaken to capture both explicit and underlying meanings grounded in participants’ own accounts rather than pre-existing frameworks ^30^.
3. Codes were clustered into provisional themes
4. Provisional themes were iteratively reviewed and refined to ensure both internal coherence and distinctiveness.
5. A thematic map was developed to capture relationships across the data, with final themes clearly defined and named in relation to the research question. This was performed based on consensus discussion with the second author to highlight any assumptions missed during the analytical process.
6. A narrative report was developed to define and explore themes with supporting extracts, in line with the Standards for Reporting Qualitative Research ^31^.

Rigour was supported through multiple strategies, including peer debriefing, use of a reflexive journal, and maintenance of an audit trail. These measures enhanced transparency and credibility, ensuring the findings were both systematically derived and aligned with the interpretivist paradigm. The lead researcher’s prior experience (as both an Irish-trained doctor who entered medical education via HPAT, and a former worker in HPAT preparation material design and delivery) and associated risk of scepticism towards aptitude testing was explicitly acknowledged and managed through reflexive journaling and regular debriefings with the research supervisor to reduce bias and prioritise participants’ perspectives.

## Results

Twelve graduates of Irish medical schools were interviewed. All entered an undergraduate medical degree after 2009. The sample was evenly balanced between males and females, and contained participants at various stages of their career, from one to ten years of postgraduate experience. Mean interview duration was 34 minutes (range: 25 – 50). Participant demographics are given Table 1.

**Table 1.** Participant demographics.

Participant	Gender	PGY	Seniority	Specialty (Subspecialty)
**P1**	F	5	Registrar	Medicine (Nephrology)
**P2**	M	2	SHO (or equivalent)	Surgery (General)
**P3**	M	1	Intern	Undifferentiated
**P4**	F	10	GP	GP
**P5**	M	5	Registrar	Medicine (Gastroenterology)
**P6**	M	10	GP	GP
**P7**	F	7	Registrar	Medicine (Nephrology)
**P8**	F	3	SHO (or equivalent)	Undifferentiated
**P9**	M	10	Consultant	Radiology
**P10**	M	7	Registrar	ED
**P11**	F	4	SHO (or equivalent)	Paediatrics
**P12**	F	8	Registrar	Surgery (Cardiothoracic)

**Figure 1.**
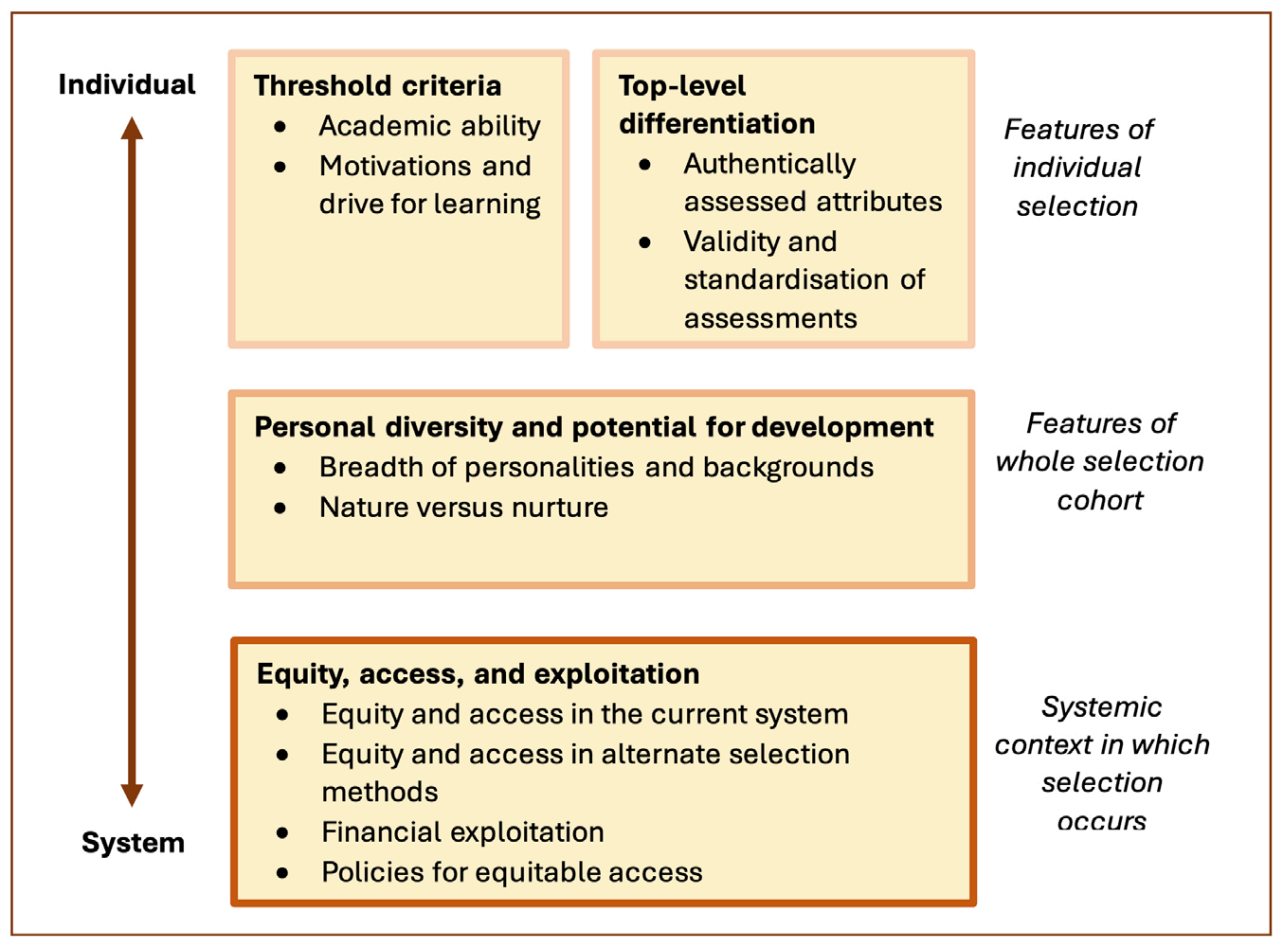
Theme map.

Participants expressed a diverse range of views regarding the state, needs, and potential alternatives to selection for medicine process in Ireland. Themes and subthemes as mapped in Figure 1 were developed from the data. The themes, as reflected in the theme map, focused on a spectrum ranging from the level of the individual applicant to the level of the system as a whole. The findings below explore each theme in depth, highlighting both consensus and contradiction across the participant responses.

### Threshold criteria as foundations of selection

When discussing what attributes are important to assess for student selection, and how these are assessed, participants made a key distinction: threshold criteria (attributes considered essential to meet a baseline standard), and additional differentiators. They described two key characteristics of threshold criteria in their relevance to student selection: [1] once met, further performance above the threshold is less relevant and should not differentiate candidates, and [2] they give an incomplete assessment of a candidate, with further differentiators being essential. This theme will deal with those attributes viewed by participants to be threshold criteria, and how they can effectively be assessed.

***Importance of academic ability and its assessment.*** Participants most consistently cited a high degree of academic ability (the capacity to process, retain, and reproduce large volumes of information) as essential for coping with the demands of a medical degree and subsequent training. The emphasis was often on managing volume rather than complexity, as P6 explained:

*“So you need to be able to absorb a lot of information and be able to do that quite quickly. There’s a perception out there that medical school is very difficult, and I'm not sure that that's always the case … Some of the work is difficult, but a lot of it is more about how much information you can retain, remember, and then apply. So I think that's something that is important.” (P6)*

However, participants almost unanimously viewed this measure as too one-dimensional, arguing that additional attributes were needed to identify effective doctors.

*“I just think like being really good at exams shouldn't be the only thing that decides it. And I think that having something to make it a bit more accessible if you're not somebody who's going to get absolutely full marks in all their exams, but that doesn't necessarily mean that you would be a bad doctor.” (P10)*

Many explicitly described the threshold nature: once a candidate was above a certain level, further differences in performance mattered less. In the context of the LC (max score 625), P7 noted:

*“I do think that north of high five hundreds, everyone's on a similar level.” (P7)*

The LC was generally considered an acceptable measure of academic assessment. Its strength in assessment was seen to be its alignment with the volumetric requirements of the medical degree programme, rather than direct subject matter relevance.

*“You need to be able to digest large volumes of information and apply them. And like, ironically enough, I think the Leaving Cert is a very good test of that.” (P6)*

In line with these views and the current structure of the Irish selection process, participants framed the LC as the core threshold criterion, with an adjunctive assessment, a role currently being filled by HPAT-Ireland, being required as a top-level differentiator. Some participants noted this to be at odds with the planned 2027 changes – removing high scoring moderation negates the key features of a threshold criterion.

*(Discussing the potential impact of 2027 changes, where 625 is the maximum LC score): “I think you're probably going to get a lot of clustering of 625 and middling HPATs” (P7)*

***Character motivations and drive for learning.*** Participants also discussed less tangible attributes, particularly motivation to seek learning and development, seen as vital for both completing medical school and remaining effective in a constantly evolving profession. Multiple participants suggested motivation could compensate for gaps in other attributes:

*“I think just having the drive to want to always be improving and always wanting to be studying. So I think overall it's less that you're born with those skills or you just innately have them. I think it's more that you need to have the type of personality where you're willing to learn those things.” (P8)*

However, P7 observed that some individuals who pass admissions screening still lack this drive, limiting their clinical growth. This in particular implies that this is an innate and desirable attribute that is currently insufficiently assessed.

*“I think you see it a lot with interns sometimes that they just don't have insight into how to get those skills or they don't have insight that those are skills that are needed.” (P7)*

Participants highlighted that assessing intrinsic motivation accurately is difficult – but the work required to perform well in the LC and HPAT often serves as a proxy indicator.

*(Discussing how to assess levels of motivation): “If you can score high in your Leaving Cert, you're motivated, you're going to have the right attitude, you're going to be a hard worker.” (P12)*

Presented less frequently was the relevance of a prospective medical student’s own moral or ethical values – while however not presented as something that could be easily or practically assessed.

*“There probably should be some level of assessment of morals or just sort of values. Just because I feel like the overarching sort of thing about medicine and being a doctor is essentially treating people who have problems. If you didn't really care about that then then it probably wouldn’t be the best thing for you.” (P3)*

Similar to academic ability, these traits were often described as a threshold criterion rather than a continuous differentiator. Possessing drive or motivation was seen as part of a larger whole – further attributes still needed to be demonstrated. This highlights the complexity of the idealised selection process described by participants: multiple thresholds must be met, yet distinguishing between candidates beyond these points remains imprecise.

### Further criteria as top-level differentiators

Participants framed further attributes as the differentiators between those participants who surpass the threshold criteria. Difficulty arose in defining and consistently assessing these attributes, particularly in relation to designing assessments that are both relevant and realistic to real-life applications. This theme will deal with these top-level differentiator criteria and their assessment.

***Authentically assessed attributes.*** Participants consistently emphasised that certain cognitive and interpersonal attributes are central to medical work, but believed these skills must be assessed authentically, in ways that reflect their real-world application rather than abstract test formats. Across the interviews, two broad domains were highlighted: clinical reasoning and problem solving, and interpersonal and communication skills. While participants viewed these as essential attributes, they were sceptical that HPAT’s time-pressured, single-best-answer format could meaningfully capture either domain.

Clinicians described problem solving, critical thinking, and the ability to work under uncertainty as “bread and butter” aspects of clinical practice. Although they acknowledged that HPAT’s logical reasoning section had surface-level relevance, they felt that multiple-choice items prevented candidates from demonstrating the actual reasoning processes they considered central to safe practice. Interview-based methods, particularly MMIs or practical scenario stations, were favoured for authenticity because they allowed candidates to articulate and adapt their thinking in real time.

*“I think one of the maybe limitations of like the aptitude tests we have is that you can't explain that reasoning. The reasoning is what you're testing, and ultimately I think reasoning is best explained and not in an MCQ format” (P7)*

*(Discussing MMIs): “Seeing how you're dealing with problems in real time, I think that would be probably the way I would prefer personally.” (P8)*

A similar pattern was identified by participants regarding interpersonal and communication skills, which they described as foundational to effective clinical work. While HPAT’s personal interactions section was understood to target these attributes, participants doubted whether the skills could be captured through decontextualised, time-pressured written vignettes. Several felt the format rewarded emotional vocabulary more than genuine interpersonal sensitivity. Similarly to the cognitive abilities described above, participants favoured the authenticity of dynamic, in person assessment methods.

*“No matter how much clinical knowledge you have, it's very hard to apply it if you can't communicate clearly with your team or with your patient.” (P8)*

*“The section on personal interactions, having them under incredible time pressure where you've got 60 seconds or 90 seconds per question. I'm not sure if that's the best way of assessing those skills. You know, in that kind of environment.” (P3)*

Non-verbal reasoning was the only domain that elicited mixed views. It was seen as relevant mainly in pattern-intensive specialties such as radiology or pathology, but of limited value as a general differentiator for medical school selection. Some even suggested that overemphasising pattern recognition might detract from broader clinical reasoning.

*“I think if you get very reliant on pattern recognition, you lose that lateral thinking, which is the reason why you're the team leader in that environment.” (P12)*

Across all three domains, participants converged on a core principle: key attributes for medical practice should be assessed in ways that meaningfully reflect their real-world application. Authenticity, contextuality, and the ability to demonstrate thought processes were considered essential, yet largely absent from HPAT’s current format.

***Validity and standardisation of top-level differentiators.*** Participants evaluated top-level differentiators primarily in terms of their validity (what they measure) and standardisation (how consistently they measure it). Across methods, they perceived persistent tensions between authenticity and fairness, with no tool achieving the ideal balance.

HPAT was generally regarded as an acceptable, if not ideal, adjunct to academic assessment. This acceptance stemmed from the unanimous view that an additional measure beyond the LC is essential, reflecting the framing of academic assessment as a necessary threshold criterion. P2 illustrated this conditional support:

*“I think based on Leaving Cert results alone, you need 625, maximum points at a minimum to get in. And so I think having an extra exam was a good idea.” (P2)*

A small number saw HPAT as appropriate for this role, but most argued that it was misaligned with the requirements of medical student selection and alternate methods would be preferred. This was put succinctly by P4, when discussing the exclusionary effect of HPAT:

*“They would be lovely, excellent doctors and then they can't get the HPAT.” (P4)*

Further, several participants considered HPAT to test a narrow and specific skillset. Even in areas theoretically well aligned with medical student selection, the questions were seen as limited in scope, particularly when considered alongside the desirability of admitting students with a broad range of skills. P10 encapsulated this view:

*“It's quite a specific exam that's trying to examine a general concept that asks very specific questions.” (P10)*

In light of these concerns about HPAT’s constructive alignment, many participants favoured the planned reduction in HPAT contribution from 2027. This was in tension with concern about removing LC moderation, and in all this framed a perception that the 2027 changes are an incomplete measure, not fully aligning with the perceived need of an appropriate threshold/top-level assessment balance.

*(Discussing the reduced contribution of HPAT score from 2027): “I think the change in the weighting might make some sense, given that I don't think that test tests exactly the things that I would want to see tested for people going into medicine.” (P8)*

One participant raised concern about interview validity, presenting interviews as a skill that many who would otherwise make good doctors do not possess, implying a lack of alignment with desired outcomes. This was otherwise unsupported, suggesting high perceived validity overall, as other methods including HPAT showed much more deeply contested, or outright negative, views on validity. However, this does point to a broader caution in the dataset: performance-based assessments, whether interviews or timed tests, risk overlooking capable candidates whose strengths emerge in less pressurised contexts.

*“So if you've got someone who doesn't perform well in an interview setting, but they might be perfectly suited to being in medical school. So yeah, I'm not sure that interviews are necessarily a way that I'd go about it” (P6)*

The issue of interview reliability was raised by many. Some feared that inconsistent interviewer engagement, for example unmotivated clinicians participating for continuous professional development benefit, would compromise integrity and fairness. Others highlighted the inherent subjectivity and influence of interviewer personality, mood, and other external factors. These concerns imply a relative strength of the LC/HPAT combination in its consistency and standardisation.

*“But then I suppose interviews also really can be quite subjective, in that it depends on your interviewer, it depends on how you are on the day, depends what's going on in your life as well.” (P2)*

Validity of personal statements was unanimously dismissed, due to perceived ease of “gaming” through external assistance, such as paid writers, family members, or generative AI. This was seen as disproportionately advantaging applicants with family in medicine or professions skilled in persuasive writing, such as law or politics. The lack of verification is also seen to predispose to idealised self-portrayals, producing homogenised submissions with differentiation of candidates. This consensus reflects a wider scepticism toward self-reported tools without robust verification.

*(Discussing personal statements): “I can't see them being of any benefit because as a 18 year old undergraduate, you're not going to write that personal statement, you're going to pay someone or you're going to get your parents to write it for you. It’s just no way it's going to be purely you.” (P5)*

### Personal diversity and potential for development

This theme deals with overarching considerations about medical student admission patterns, which participants viewed as important but cannot be assessed under a single attribute.

***Breadth of personalities and backgrounds.*** It was strongly expressed by participants that while important to assess specific criteria, admissions should accommodate a diverse range of backgrounds and personality types. As described in “Validity and standardisation of top-level differentiators”, HPAT was seen to go against this desirability for broad personalities and backgrounds.

*“You know, there are many different types of doctors, and you need many different types of people for those roles. I'm a GP, like I wouldn't do very well as a vascular surgeon and I'm sure there's lots of vascular surgeons that wouldn't do very well in a GP environment. So having a very narrow bandwidth for those that enter medical school, you're going to get a very narrow output then in terms of the type of people that you get and at the end of the day medical school is trying to fill not just vascular surgeons or general practitioners, but also public health doctors and people with a research brain or people who want to work in emergency departments or anaesthetics or radiology.” (P6)*

***Nature versus nurture.*** A recurring theme was whether desirable attributes are intrinsic and appropriate to assess at entry, or largely acquired during young adulthood and training – raising doubts about validity if measured too early. Views were mixed, but certain patterns were identified: motivation for learning and the ceiling of academic ability were largely seen as intrinsic, while interpersonal and communication skills were more often considered underdeveloped at the age of admission and thus unfair to measure at this stage. This distinction has important implications for selection design: if key qualities are only partially formed, overemphasis in admissions risks both inequity and inaccurate prediction of future performance. These findings support a more holistic admissions approach – one that balances measurement of relatively stable traits with recognition that other desirable attributes can and should be cultivated during training – rather than a rigid, criteria-linked system that may prematurely reward privilege and penalise late development.

*(Discussing interpersonal skills as an innate vs developable attribute): “If you're an awkward 18 year old, of course, talking to someone's your worst nightmare. Whereas when you grow up a bit and you've been in the situations and you feel confident in your knowledge and abilities, you're more comfortable in that kind of interpersonal space”*

### Equity, access, and exploitation

Participants presented equity as a central concern, shaping both critiques of the current system and hesitation about proposed alternatives. Participants described inequities as systemic rather than confined to any single tool, with consequences for both individual candidates and the health system at large. This theme explores the mechanisms of these perceived inequities and the potential paths and limitations to addressing them.

***Equity and access in the current system.*** Participants consistently viewed HPAT as inequitable, with costs for sitting, resits, and coaching disproportionately benefitting wealthier applicants. Further inequities were attributed to the presence of strong parental support and resource availability in more advantaged schools. This consensus underscores the perception that HPAT does not merely measure aptitude but also reflects and reinforces pre-existing socioeconomic advantage, entrenching inequality at the gateway to medical training.

*“You're really helping your chances if you have the money to invest into it and do the prep courses. There probably is a sort of unfairness in the way if you can't afford them, your chances of doing better I think are lessened, you know, not that you can't do well, but I think your chances are lessened.” (P3)*

However, participants largely valued the relative anonymity of the LC/HPAT combination. Compared with in-person assessments or personal statements, there was seen to be lower scope for bias based on family connections, ethnicity, gender, appearance, or background. Furthermore, it was noted that engagement with the material and sustained effort, which ultimately rests with the individual and cannot be purchased, remained essential to success. This presents an interesting dilemma: the system rewards motivation for learning as a threshold criteria, yet if HPAT/LC falls short in capturing other relevant attributes, this single quality becomes disproportionately weighted in selection outcomes.

*(Discussing the HPAT/LC combination favouring motivation for learning): “That kind of determination, if someone really wants it, then they'll do whatever they can. Like I suppose you might have someone that might not be the best academically, but they'll work as hard as they can so that they'll do as well to get the jobs that they want at the end of the line.” (P1)*

***Equity and access in alternate selection methods.*** Non-anonymised tools, particularly interviews, raised concerns among participants. Two main risks were identified: the influence of overt personal biases (based on ethnicity, gender, appearance, or other visible characteristics), and the potential for nepotism – the latter regarded as entrenched in Ireland, particularly within the small medical sphere. These concerns were often framed through the lens of participants’ own experiences with postgraduate job and training programme interviews, where they perceived such inequities to be already present. This suggests that, for many, fears about equity in undergraduate selection are shaped by their broader understanding of medical career progression.

*(Discussing potential biases in selection methods): “I guess when you're seeing them in person, it's kind of a bit more confronting because you can see quite plainly if it's someone of a different race, someone with an accent, a woman, someone maybe has a few more piercings, things like that. It's more plain and it's more in your face.” (P8)*

*(Discussing the potential for nepotism in student selection): “With interviews especially in a country like Ireland, it's very small, and it's rife for nepotism and not even conscious nepotism … if you've got panels and you've got a kid who's Mom or Dad is a is a high flying consultant or high flying GP” (P6)*

Methods based on presentation or discussion of prior experiences, such as personal statements or CV-based interview questions, were seen to inherently advantage those whose family or financial background has enabled extracurricular activities, volunteering, or clinical observerships – opportunities less accessible to disadvantaged applicants. This adds another layer of inequity, compounding concerns about bias and nepotism.

Financial inequity in alternate selection methods was also acknowledged by all, with differing views on how its influence might compare to the HPAT/LC combination. Participants felt that performance-based assessments, including interviews and personal statements, would be susceptible to the same dynamic currently seen: proliferation of paid preparation resources, and corresponding inflation of performance standards to the point where such preparation is perceived as essential. This phenomenon is closely intertwined with the issue of financial exploitation.

*(Discussing equity in relation to interviews): “I think that's something else that can be prepped for … I'm not sure if it would improve that inequity drastically.” (P9)*

Taken together, these perspectives indicate that even methods theoretically capable of capturing desirable attributes risk being undermined by the structural realities of a small, interconnected professional community, making genuine equity challenging to secure in practice.

***Financial exploitation.*** Participants expressed tension between the concepts that selection methods are inherently financially biased, and that they only develop such bias through external exploitation. They viewed it as highly undesirable that third-party private organisations can establish profit-driven preparation services, for two principal reasons: firstly, the exploitation of applicants and their families, placing them under significant financial pressure; and secondly, the inflation of performance standards, creating a self-perpetuating dependence on paid preparation. This dynamic was seen to shift selection from a test of aptitude to a test of access to resources. P11 describes this shift clearly:

*‘People who do well in the HPAT have done HPAT preparation courses, which are phenomenally expensive. They have come from a position where they are privileged enough to be able to do those. So I do not necessarily think that the people who do well in the HPAT are always the people who have that natural ability or who have this academic brilliance.” (P11)*

***Policies for equitable access.*** Many participants raised other wishes relating to fairer access to medical education. Among these was concern that the current reality of undergraduate medical admissions in Ireland is an over-representation of one socioeconomic and ethnic group, discouraging individuals from outside these groups from pursuing medical careers, perpetuating a workforce unrepresentative of the population it serves. Proposed solutions to this were the introduction of admissions quotas of individuals from underrepresented socioeconomic or ethnic groups.

*“I think bringing in a quota would be something that I'd be interested in doing. And so that's I suppose international quotas or migrant quotas and then also maybe people from different strands of society. You know, so that if you're coming out of a deep end school that you've a slightly higher chance, if you're willing to apply yourself and meet some of those entry criteria, that you have a chance of getting in.” (P6)*

Also stressed was the value of multiple parallel pathways into medicine and point-reduction schemes for disadvantaged applicants, as ways to reduce homogeneity in admissions. Such approaches were seen to address socioeconomic and ethnic overrepresentation while also favouring diversity of personality types and backgrounds, highlighting the strong desire among participants for more equitable access to medical education.

## Discussion

### Overview

This study provides new in-depth information about the views of a new generation of doctors on selection for medicine. Their dual role as current stakeholders working in the health system and former HPAT-Ireland candidates provides unique insight. Participants conceptualised selection as a two-stage process: first, meeting threshold criteria such as academic ability, and second, distinguishing between candidates through higher-order skills. While the LC was seen as an appropriate threshold, HPAT-Ireland was widely criticised as a flawed differentiator. The following sections explore these themes in relation to international evidence and policy implications.

### Threshold criteria

Participants’ emphasis on academic ability aligns with international empirical evidence that it outperforms other assessment methods in predicting medical school performance, retention, and progression ^1, 3^. However, clear limitations are also seen in the over-dependence on academic assessment in student selection – namely its attenuating predictive power over time ^32^, and its lack of insight into other key skills and cognitive domains ^33, 34^. These limitations reinforce its role as a threshold criterion rather than a sufficient standalone measure, echoing participants’ perspectives. Debate remains over what should complete the assessment picture as top-level differentiators – what McManus et al term the “dark variance” of student selection ^33^, which participants saw as a spectrum of empathy, communication, and reasoning. This context aligns with some participants’ concern that the 2027 reforms (removing moderation of high LC scores) may overemphasise academic results and weaken the current threshold-based balance.

Motivation and ethical orientation were regarded as essential threshold attributes. Intrinsic motivation is known to predict engagement, persistence, and academic outcomes ^35^. Self-Determination Theory holds that motivation is fostered in supportive environments ^36, 37^. This convergence of theory and participant experience suggests that while motivation matters, it may be more effectively nurtured within medical education than reliably assessed at entry. While not directly assessed, participants saw the work required to perform in the LC/HPAT combination as a proxy indicator of motivation. Values such as empathy and integrity are central to frameworks like CanMEDS and GMC Outcomes for Graduates ^38, 39^, but remain challenging to assess fairly. Self-report tools are vulnerable to coaching and bias, while SJTs and ethics-focused MMI stations show only modest validity ^40– 42^.

### Top-level differentiation

Participants identified critical thinking, problem solving, and communication skills as the most meaningful differentiators once academic thresholds are met, consistent with graduate outcome frameworks ^38, 39^. There is varying empirical support for the use of these attributes in student selection - reasoning and problem-solving ability predict early academic success but lose value over time ^15, 33^. Beyond academic performance, evidence shows that shortcomings in analytical reasoning at higher professional levels are linked with clinical errors ^43– 45^, clearly showcasing their importance. Communication skills, meanwhile, have been consistently linked to clinical effectiveness, teamwork, patient satisfaction, and reduced error rates ^46, 47^.

Assessment methods vary in their ability to capture these attributes. Aptitude tests such as HPAT reduce complex processes to abstract multiple-choice tasks, limiting ecological validity, or real-world applicability ^1^ – evidence directly echoing participants’ lack of faith in the authenticity of HPAT’s assessment. Considering the above evidence of the importance of these attributes, this may reflect an opportunity for significant improvement in selection processes in Ireland.

In contrast, MMIs and SJTs show moderate validity for academic and clinical performance ^5, 48, 49^, likely due to their closer resemblance to real clinical challenges and contexts. Participants’ preference for MMIs over HPAT mirrors this evidence base: both emphasise contextualised judgement rather than abstract reasoning.

Taken together, these findings and the literature converge on a consistent message: more authentic assessment of higher level differentiators is desirable, but challenge lies in designing tools to assess these skills realistically, reliably, and equitably – whereby Irish standards and international best practice are seen to diverge.

### Equity, access, and exploitation

Equity concerns were strongly cross-cutting in discussion of all assessment attributes and tools. Participant views on financial inequity linked to HPAT mirror evidence that aptitude tests such as UKCAT and UMAT are influenced by socioeconomic status ^50^ – with particular evidence that those receiving aptitude test coaching may in fact perform worse in medical school examinations ^51^, again supporting the view that issues of inequity affect the overall performance of the system as well as the individual applicant. There is similar evidence that other assessment methods – notably structured interviews and MMIs – are subject to the same concerns of paid coaching, inflated scores, and subsequent financial inequity ^1^.

Concerns about nepotism and personal biases in non-anonymised selection methods are well supported by international evidence that traditional interviews are subject to poor inter-rater reliability and inequity based on gender, ethnicity, and socioeconomic status ^1, 52^. These inequities are not confined to admissions but mirror the wider patterns of differential attainment across training and career progression identified by Woolf ^52^, reinforcing that inequitable entry criteria can entrench systemic disadvantage throughout the profession. This illustrates how individual experiences of bias in Irish admissions resonate with international evidence of systemic inequity across medical careers, and implies admissions equity to be inseparable from building a sustainable, representative medical workforce.

Participants advocated structural approaches such as quotas or parallel entry pathways, echoing evidence that targeted reforms can widen access when supported by institutional commitment and engagement ^53^. Improving diversity was seen not only as beneficial for individual fairness but as a system advantage, consistent with studies linking diversity to stronger clinical performance, patient outcomes, and health-system sustainability ^54^.

Viewed collectively, participant experiences and international evidence highlight equity challenges as systemic rather that confined to tool selection alone. This highlights the risk that reforms limited to reweighting or modifying individual assessments act as technical fixes, leaving deeper inequities intact. Addressing equity in admissions therefore requires coordinated, system-level approaches that prioritise transparency, consistency, and safeguards against exploitation, rather than isolated adjustments to individual tools.

### Implications for 2027 changes

The findings carry direct implications for ongoing reforms to Irish medical school admissions. Planned 2027 changes aim to reduce HPAT weighting relative to the LC. While this may alleviate some inequities, participants’ critiques suggest that this is an incomplete rather than comprehensive fix, and deeper concerns remain about the role of aptitude testing and the weakening of the use of the LC as a threshold criterion. These findings align with international debates emphasising that equity is difficult to achieve through isolated reforms. Evidence from the UK and Australia shows that reweighting or rebranding aptitude tests has had little effect on widening access, with underlying socioeconomic disparities persisting despite policy changes ^18, 55^. Systematic reviews similarly conclude that isolated or incremental adjustments risk being perceived as pragmatic “technical fixes” rather than substantive solutions ^1^.

Beyond this evidence, the concept of political validity ^10^ suggests that participant critiques themselves may reflect wider opinions that will undermine the 2027 changes. Reforms should therefore consider a broader recalibration: embedding dynamic and authentic assessments of differentiator criteria (such as MMIs or SCs), while taking measures to mitigate bias and nepotism (such as transparent and standardised procedures and robust assessor training). Stakeholder engagement, especially with those who have experienced the system directly, will be essential for legitimacy and sustainability.

### Strengths and limitations

This study has several strengths. It provides the first qualitative exploration of the “HPAT generation,” offering insights unavailable from policymakers, educators, or literature alone. Rich narrative data and reflexive thematic analysis enabled an in-depth exploration of perspectives. Rigorous quality assurance, including peer debriefing and audit trails, enhanced trustworthiness.

However, limitations must be acknowledged. First, recall bias is possible, as participants’ own admissions experiences ranged from six to fifteen years earlier. Second, the sample was confined to doctors trained in Ireland, limiting transferability beyond this context. Finally, the researcher’s positionality as a peer may have shaped data interpretation (specifically through scepticism towards aptitude testing), though reflexivity was employed to mitigate this. These limitations suggest caution in generalising but do not diminish the significance of the findings for Irish policy debates.

### Implications for future research

Future research should move beyond predictive validity of academic performance toward longitudinal evaluation of how admissions criteria relate to clinical competence, professional behaviours, patient care and outcomes, and workforce diversity. Further stakeholder perspectives – such as educators, patients, and applicants – should be incorporated to strengthen political validity and sustainability of reforms. Analysis of admissions beyond 2027 are essential to assess if equity and diversity goals are being met in practice. Longitudinal monitoring of how these students perform academically and in professional practice can provide further evidence on the need to reformat selection methods in future. Finally, innovative, multi-method approaches that blend fairness, reliability, and feasibility are needed to ensure selection systems capture not only who can succeed in medical school, but who can best serve as the future of the medical profession.

## Conclusion

This study highlights enduring tensions seen in medical student selection between validity and equity. Doctors who entered through HPAT-Ireland recognised the need for rigorous academic thresholds but challenged the appropriateness of HPAT as a differentiator. They advocated for the adjunctive use of more authentic assessments, with many directly advocating for practical, in person methods such as MMIs or SCs, while aware of major inequity challenges to overcome at a systems level in their hypothetical implementation. Safeguards against these challenges, such as robust rater training and transparent scoring systems, are seen as essential. Overall, the alignment between participant perspectives and international evidence reinforces the need for reforms that balance validity and fairness. Future policy must confront these trade-offs transparently to maintain stakeholder trust.

## Ethical approval

Ethical approval was granted by the University College Cork Social Research Ethics Committee (SREC) on March 6th 2025, with log number 2025-008.

## Consent

Written informed consent for the recording, analysis, and publishing of participants’ statements was obtained from the participants.

## Data Availability

Due to the potentially identifiable nature of qualitative interview data in a small medical community, ethical approval was granted on the grounds of only short, anonymised excerpts being included in the article. Participants consented to the use of excerpts only; therefore, full transcripts cannot be shared. Additional de-identified extracts may be provided upon reasonable request, by contacting the corresponding author ( 117369726@umail.ucc.ie), and subject to approval by the University College Cork Social Research Ethics Committee. Requests must specify the data required, purpose of use, intended audience, storage plans, retention period, and data-protection measures. Zenodo. Data Repository - A Qualitative Study of a New Generation of Irish-Trained Doctors' Views on Selection for Medicine. https://doi.org/10.5281/zenodo.18217841 ^56^. The project contains the following underlying data: Extended Data File 1 – Interview guide. (A copy of the semi-structured guide used for interviews) Extended Data File 2 – SRQR. (A copy of the Standards for Reporting Qualitative Research checklist, in line with which this study was reported) Extended Data File 3 – PIL:Consent. (A copy of the participant information leaflet and consent form with which participants were invited to the study) MEP Figure 1. (A copy of the theme map developed during this study) MEP Table 1. (A copy of the table showcasing participant demographics in this study) Data is available under the Creative Commons Attribution 4.0 International license. This study was reported in line with the Standards for Reporting Qualitative Research. The completed SRQR checklist is provided as extended data (File 2).
