# Supplementary material for: A Qualitative Study of a New Generation of Irish-Trained Doctors’ Views on Selection for Medicine
Source: MedEdPublish (2016). 2026 Jan 26;16:3. [Version 1] doi: 10.12688/mep.21457.1 (PMC13022560; doi:10.12688/mep.21457.1)
Supplement: Peer review history

# Reviewer report: Reviewer response for version 1

**DOI:** 10.21956/mep.22983.r45641

**Published:** 2026-03-27

**Authors:** Chitra Subramaniam

**Affiliations:** 1 Learning and Development, AO North America (Ringgold ID: 457098), Wayne, Pennsylvania, USA

The article clearly articulates the aims and the relevant study design, results and conclusions.  The steps undertaken in the qualitative assessment of the responses provided by the interviewees are robust and clearly support the aim of the study.  

 One aspect that is missing and not explained anywhere in the article is the development of the interview guide.  Since it was a structured interview, the development of appropriate and valid questions that address any orientation of the interviewees, bias and or unrelated questions.  I did not see the process involved in the development of the structured interview.  While the themes and participant profile is included, the structured interview questions are not included and there is no description of how it was developed, reviewed and validated. 

 Overall, the research processes are relevant and technically sound.

Have any limitations of the research been acknowledged?

Yes

Is the work clearly and accurately presented and does it cite the current literature?

Yes

If applicable, is the statistical analysis and its interpretation appropriate?

Yes

Are all the source data underlying the results available to ensure full reproducibility?

Yes

Is the study design appropriate and is the work technically sound?

Yes

Are the conclusions drawn adequately supported by the results?

Yes

Are sufficient details of methods and analysis provided to allow replication by others?

Yes

Reviewer Expertise:

Educational research including both quantitative and qualitative, and mixed methods

I confirm that I have read this submission and believe that I have an appropriate level of expertise to confirm that it is of an acceptable scientific standard.

# Reviewer report: Reviewer response for version 1

**DOI:** 10.21956/mep.22983.r45521

**Published:** 2026-03-26

**Authors:** Tayseer M Mansour

**Affiliations:** 1 Medical Education, Suez Canal University Faculty of Medicine (Ringgold ID: 68833), Ismailia, Ismailia Governorate, Egypt; 2 Family & Community Medicine and Medical Education, Taibah University Faculty of Medicine (Ringgold ID: 123307), Medina, Al Madinah Province, Saudi Arabia

This manuscript reports an interpretivist qualitative study in which twelve Irish-trained doctors who entered medicine after 2009 the so-called "HPAT generation" were interviewed about their views on medical student selection. Using reflexive thematic analysis following Braun and Clarke's six-phase framework, the authors identified four themes: threshold criteria as foundations of selection; further criteria as top-level differentiators; personal diversity and potential for development; and equity, access, and exploitation. The central argument is that this cohort supports a two-stage selection system but is sceptical of HPAT-Ireland's capacity to fulfil its intended differentiating role, and that the planned 2027 reforms are an incomplete rather than comprehensive solution.

 This is a timely, relevant, and generally well-executed study that makes a genuine contribution to the literature on medical student selection. The "HPAT generation" constitutes a uniquely positioned stakeholder group one that has lived experience of both the selection process and subsequent clinical training and their perspectives have been largely absent from the existing evidence base. The manuscript is clearly written, logically structured, and demonstrates a commendable degree of methodological transparency, including explicit acknowledgement of the lead researcher's positionality. The literature review is appropriately scoped and engages with key international evidence.

Have any limitations of the research been acknowledged?

Yes

Is the work clearly and accurately presented and does it cite the current literature?

Yes

If applicable, is the statistical analysis and its interpretation appropriate?

Not applicable

Are all the source data underlying the results available to ensure full reproducibility?

Yes

Is the study design appropriate and is the work technically sound?

Yes

Are the conclusions drawn adequately supported by the results?

Yes

Are sufficient details of methods and analysis provided to allow replication by others?

Yes

Reviewer Expertise:

Medical education

I confirm that I have read this submission and believe that I have an appropriate level of expertise to confirm that it is of an acceptable scientific standard.

# Reviewer report: Reviewer response for version 1

**DOI:** 10.21956/mep.22983.r45452

**Published:** 2026-03-19

**Authors:** Richard Hays

**Affiliations:** 1 James Cook University, North Queensland, Australia

Context. This article reflects a fascinating, long term debate about selection processes for undergraduate medical education. Many programs use a variety of combinations of academic track record and aptitude tests, often with some form of interview (from multi-station to more traditional formats). The research around all has produced rather unclear results, with little evidence of construct or predictive validity. Selection methods can provide logistic challenges and there is a strong appetite for finding a better way. A particular strength is the description of the history and context in Ireland, along with international trends and evidence.

 Writing and structure. The paper is well-written and clear.

 Literature review. This is sound, providing a useful brief summary of the relevant literature.

 Methods. I have some concerns about both the conceptual basis of the study and the premise that results explain proposed changes to selection policy and processes in Ireland. 15 years is a long time and societal changes are likely much more influential than the introduction of HPAT as an influence on thinking. The time period in question has been one of rapid social change - the impact of social media on communication and thinking is one example of potential confounders. This constrains interpretation of interview transcripts of 12 people selected non-systematically. Potential recall bias is acknowledged but not the potential for 'survivor’ or 'success’ bias - that is, the interviewees were selected in part on their HPAT performance. This may affect their views, perhaps more in favour of the system that selected them. Then, using this narrow, perhaps not so sound base, suggesting that the proposed change in HPAT use is justified by this study may be an over-reach. The time frame suggests that the change was on the agenda for some time - was this study done to inform or justify thinking? The article would be much more balanced if the broader and highly complex contextual evolution were to be acknowledged and softening of this small qualitative study as the provider of evidence to support change. Selection remains a messy field and this should be acknowledged rather trying to simplify decisions on methods and processes. It is easy to support selection processes that produce a broad range of personalities, styles, altitudes and interests but so far very little predicts any particular outcome. It remains unclear exactly what HPAT-like tests actually measure and their use has not been shown to do much more than apparently make one step in shortlisting more ‘objective’. Addressing these issues would add value to the article for those seeking to explore selection in greater depth.

 Likely audience. Of potential interest to all involved in primary medical education.

Have any limitations of the research been acknowledged?

Partly

Is the work clearly and accurately presented and does it cite the current literature?

Yes

If applicable, is the statistical analysis and its interpretation appropriate?

Not applicable

Are all the source data underlying the results available to ensure full reproducibility?

Yes

Is the study design appropriate and is the work technically sound?

Partly

Are the conclusions drawn adequately supported by the results?

Partly

Are sufficient details of methods and analysis provided to allow replication by others?

Yes

Reviewer Expertise:

curriculum and assessment design, evaluation and quality assurance

I confirm that I have read this submission and believe that I have an appropriate level of expertise to confirm that it is of an acceptable scientific standard, however I have significant reservations, as outlined above.

# Reviewer report: Reviewer response for version 1

**DOI:** 10.21956/mep.22983.r45431

**Published:** 2026-03-09

**Authors:** Rhoda Meyer

**Affiliations:** 1 Stellenbosch University, Stellenbosch, South Africa

The introduction is well written, providing a background of student selection for undergraduate medical degree programmes. The authors attempt to delineate the gap and highlight the value of this study for adding to the field.

 The methodology is well aligned to answer the research question.

 This study fills an important gap by capturing the perspectives of the "HPAT generation," offering insights that are both timely and relevant given upcoming policy reforms.

 The thematic analysis is rich, with illustrative participant quotes that enhance credibility. The discussion connects findings to broader debates in medical education and selection validity. While this is a well written submission, it could be strengthened by:

 Expanding on how findings might inform practical implementation of selection reforms beyond policy (e.g., implications for admissions committees).

 A brief reflection on limitations of sample size and representativeness could strengthen transparency, even though qualitative sufficiency is acknowledged.

 The lead researcher’s prior involvement in HPAT preparation materials is acknowledged, but the reflexivity section could be expanded, specifically on how positionality shaped interview dynamics and interpretation.

Have any limitations of the research been acknowledged?

Yes

Is the work clearly and accurately presented and does it cite the current literature?

Yes

If applicable, is the statistical analysis and its interpretation appropriate?

Not applicable

Are all the source data underlying the results available to ensure full reproducibility?

Yes

Is the study design appropriate and is the work technically sound?

Yes

Are the conclusions drawn adequately supported by the results?

Yes

Are sufficient details of methods and analysis provided to allow replication by others?

Yes

Reviewer Expertise:

Health Professions Education, curriculum development, faculty development

I confirm that I have read this submission and believe that I have an appropriate level of expertise to confirm that it is of an acceptable scientific standard, however I have significant reservations, as outlined above.
